# Micromechanical Modelling of the Influence of Strain Ratio on Fatigue Crack Initiation in a Martensitic Steel-A Comparison of Different Fatigue Indicator Parameters

**DOI:** 10.3390/ma12182852

**Published:** 2019-09-04

**Authors:** Benjamin Josef Schäfer, Petra Sonnweber-Ribic, Hamad ul Hassan, Alexander Hartmaier

**Affiliations:** 1 Robert Bosch GmbH—Corporate Sector Research and Advance Engineering, 71272 Renningen, Germany; 2 Interdisciplinary Centre for Advanced Materials Simulation, Ruhr-Universität Bochum, 44801 Bochum, Germany (H.u.H.) (A.H.)

**Keywords:** martensitic steel, fatigue crack initiation, crystal plasticity, fatigue indicator parameters

## Abstract

Micromechanical fatigue lifetime predictions, in particular for the high cycle fatigue regime, require an appropriate modelling of mean stress effects in order to account for lifetime reducing positive mean stresses. Focus of this micromechanical study is the comparison of three selected fatigue indicator parameters (FIPs), with respect to their applicability to different total strain ratios. In this work, investigations are performed on the modelling and prediction of the fatigue crack initiation life of the martensitic high-strength steel SAE 4150 for two different total strain ratios. First, multiple martensitic statistical volume elements (SVEs) are generated by multiscale Voronoi tessellations. Micromechanical fatigue simulations are then performed on these SVEs by means of a crystal plasticity model to obtain microstructure dependent fatigue responses. In order to account for the material specific fatigue damage zone, a non-local homogenisation scheme for the FIPs is introduced for lath martensitic microstructures. The numerical results of the different non-local FIPs are compared with experimental fatigue crack initiation results for two different total strain ratios. It is concluded that the multiaxial fatigue criteria proposed by Fatemi-Socie is superior for predicting fatigue crack initiation life to the energy dissipation criteria and the accumulated plastic slip criteria for the investigated total strain ratios.

## 1. Introduction

Martensitic high-strength steels are distinguished by a high strength to weight ratio, an excellent weight and cost-effectiveness as well as a more environmental friendly energy balance than aluminium alloys. These attributes are of particular importance in today’s challenges such as the design of sustainable transportation systems [1]. Among these martensitic high-strength steels, the quenched and tempered SAE 4150 plays a pivotal role due to its excellent strength and toughness. These specific material properties are directly related to the unique hierarchical martensitic microstructure being an assembly of of laths, blocks and packets [2,3]. Despite these remarkable material properties, the components produced from this class of material are prone to a high risk of fatigue failure during service life. A fundamental prerequisite for the prediction of components fatigue life is the adequate modelling of the influence of varying load ratios on the cyclic fatigue behaviour due to the lifetime reducing effects of positive mean stresses and strains [4]. Future advanced fatigue life estimation tools should, therefore, predict mean stress effects on fatigue life precisely.

Experimental investigations on low and medium carbon-alloyed martensitic high-strength steels have been focused on several fields of research, in the last decades. Fundamental investigations on the morphology and crystallography enabled a profound understanding of the hierarchical architecture of lath martensite. During the diffusionless, shear-dominated transformation from austenite ($\gamma^{\prime }$ phase) to martensite ($\alpha^{\prime }$ phase), 12 up to 24 crystallographic variants can be formed from a single prior austenite grain (PAG) with the Nishiyama–Wassermann and Kurdjumov–Sachs orientation relationship, respectively [2,3,5]. The deformation behaviour of lath martensite structures was extensively studied e.g., by Mine et al. [6], Du et al. [7] and Shibata et al. [8] with respect to the effectiveness of grain boundaries within the hierarchical morphology of martensite. Accordingly, block and packet boundaries represent effective barriers for dislocation motion and plastic deformation transfer, respectively. The effects of mean stresses on fatigue life have been long studied, as for example in the early fundamental works of Gerber and Goodman, cited in Shijve [9]. Mean stress and mean strain effects become relevant and reduce fatigue life for elastic bulk material response as it is in the high cycle fatigue (HCF) regime [4]. An increase of the mean stress or mean strain (at constant stress or strain amplitude) yields to an increase of the maximum stress level and consequently to an increase of the driving forces for fatigue crack initiation (FCI) and growth [9]. In contrast, in the low cycle fatigue (LCF) regime of strain-controlled fatigue experiments with elasto-plastic material response, increased mean strain levels cause mean stress relaxation in early life. Consequently, the LCF life will not be appreciably affected by the superimposed mean strains in contrast to fully reversed tests [4]. In the past, a variety of martensitic high-strength steels were subject of experimental investigations about the influence of mean strains on the total fatigue life. Koh and Stephens [10] and Wehner and Fatemi [11] investigated comparable quenched and tempered medium carbon-alloyed steels and showed the detrimental effect of tensile mean strains on fatigue life for small applied total strain amplitudes in the HCF regime. However, there are only few consistent fatigue crack initiation data sets for the considered material at total strain-controlled conditions in literature available. Therefore, new fatigue tests were performed in this study.

The total fatigue life of polycrystalline materials can be decomposed into two fundamental stages of fatigue represented by macroscopic fatigue crack initiation and macroscopic fatigue crack propagation [12,13,14,15]. From a microscopic point of view, the macroscopic fatigue crack initiation stage can be further subdivided into the stages of fatigue crack nucleation, microstructurally short crack (MSC) growth and physically short crack (PSC) growth [12,16]. The nucleation period represents an early stage of fatigue life [17]. Thereby, localisation and accumulation of irreversible plastic slip are one of the primary driving forces for fatigue crack nucleation [13]. Both fatigue crack nucleation and micostructurally short crack growth are directly influenced by the local microstructural attributes, such as grain size and orientations, and reveals consequently a significant microstructural dependency [13,16]. Depending on the strength and the loading conditions of steels, the FCI life (nucleation life + microstructurally short crack life) may account for the majority of the total fatigue life, in particular in the HCF regime [14,18,19,20]. Due to the enormous experimental effort on FCI investigations on martensitic steels, they have received less attention than studies on crack propagation. In order to overcome this, micromechanical modelling techniques incorporating representative volume elements (RVE) and crystal plasticity models on the crystal scale provide powerful techniques to investigate the fatigue phenomena mechanisms in detail [16,21,22,23].

For the micromechanical prediction of fatigue crack nucleation, the local mechanical fields are used to determine mesoscopic FIPs representing driving forces for fatigue crack initiation and growth [16]. There are a variety of different FIPs correlating different micromechanical quantities to these surrogate measures for fatigue, e.g., the shear based accumulated plastic slip, the multiaxial fatigue criteria proposed by Fatemi and Socie [24] and different energy based criteria. Manonukul and Dunne [25] investigated the LCF and HCF life of a nickel alloy C263 by correlating the accumulated plastic slip with the cycles required for FCI. Based on the Tanaka-Mura model, Brückner-Foit and Huang [26] studied the heterogeneous stress distributions with an elasto-plastic orthotropic material model and predicted the crack densities in the LCF life of a martensitic steel with a good agreement to experimental results. Using an energy dissipation criteria, Cruzado et al. [22] predicted the FCI cycles for IN718 alloy for different strain ratios. The reported results were in good agreement with experimental results for both strain ratios, therefore it is assumed that the energy dissipation criteria is well suited for predictions of FCI results at different strain ratios. A comparison of the accumulated plastic strain and an energy dissipation criteria was performed by Sweeney et al. [23] for a CoCr stent material for one loading level. The corresponding results indicated, that the energy dissipation criteria shows a higher sensitivity to local stress variations than the accumulated plastic slip. A lath martensitic microstructure under fully reversed fatigue loading was also investigated micromechanically by using a shear based critical plane FIP and the Tanaka-Mura model by Briffod et al. [27]. Gillner and Münstermann [28] developed a micromechanically informed extreme value distribution density based fatigue model for the prediction of HCF life. The grain averaged accumulated plastic slip is used for the calculation of the fatigue crack nucleation life. The stages of MSC, PSC and long crack regime are modelled by an extreme value distribution density approach which has to be calibrated by experimental HCF data. The stress-controlled model shows a good agreement with experimental results for different stress ratios. However, in addition to the LCF experiments for the calibration of the crystal plasticity model, further HCF experiments are required to calibrate the extreme value distribution density approach for the propagation regime. Recently, Chen et al. [29] compared experimentally observed fatigue crack nucleation sites with predicted nucleation sites by the accumulated plastic slip, the Fatemi-Socie metric, an energy dissipation criteria and a stored energy density criterion for a nickel alloy under reversed loading conditions. They concluded, that the local stored energy density metric incorporating the density of geometrically necessary dislocations reveals the best agreement to the experimentally observed nucleation sites. However, this localisation metric requires the application of a non-local crystal plasticity model for the calculation of the geometrically necessary dislocations. There have been numerous further investigations on FCI predictions by different FIPs. However, a comparison of different types of FIPs for martensitic high-strength steels under different loading conditions is still missing in the available literature.

The purpose of the present study is the micromechanical prediction of fatigue crack initiation life for the martensitic high-strength steel SAE 4150 for two different applied total strain ratios, R${}_{\varepsilon }=-1$ and R${}_{\varepsilon }=0$, whereby the total strain ratio R${}_{\varepsilon }=\varepsilon_{t,min}/\varepsilon_{t,max}$ is defined by the ratio of the minimum applied total strain $\varepsilon_{t,min}$ and the maximum applied total strain $\varepsilon_{t,max}$ during a cycle. For this purpose, a comparison of three selected different FIPs, accumulated plastic slip, Fatemi-Socie and an energy dissipation criteria, concerning their applicability at varying loading conditions is presented in the present study.

The paper is structured as follows: first, in Section 2 the martensitic high-strength steel SAE 4150 is characterised with respect to microstructural properties as well as to experimental LCF and HCF behaviour. Next, the multi-scale modelling strategy incorporating the generation of synthetic microstructures, the constitutive model and the different fatigue indicator parameters are presented in Section 3. Results of the micromechanical fatigue investigations are presented and discussed in Section 4. Finally, the main conclusions are summarised in Section 5.

## 2. Material and Experimental Investigations

The material investigated in this study is the quenched and tempered martensitic high-strength steel SAE 4150 (German designation: 50CrMo4). The chemical composition of the material which was determined by spark emission spectroscopy is given in Table 1. Further fundamental monotonic mechanical properties are the Young’s modulus E = 208 GPa, the surface hardness of 39 HRC, the yield strength $\sigma_{f}=1075$ MPa, the ultimate tensile strength $\sigma_{u}=1178$ MPa and an elongation of 13.8%. This section focuses on the characterisation of the microstructure as well as the corresponding fatigue properties of the material.

### 2.1. Microstructure Characterisation

The underlying martensitic microstructure was analysed with respect to microstructural morphology, corresponding prior austenite grain morphology and crystallographic properties by means of light optical microscopy (LOM, Leica MZ16, Leica Camera, Wetzlar, Germany) and high-resolution scanning electron microscopy (SEM, Zeiss Merlin, Zeiss, Oberkochen, Germany) in combination with automated electron backscatter diffraction (EBSD, Oxford Aztec, Oxford Instruments, Abingdon, UK).

**Figure 1:**
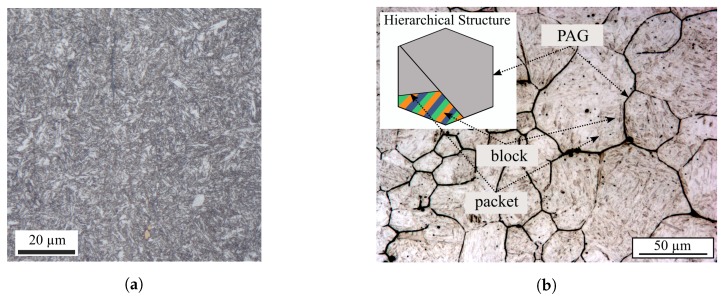
Light optical micrographs of the SAE 4150 microstructure. (**a**) Lath martensitic microstructure by nital etching. (**b**) Etching of the prior austenite grain boundaries by a picric acid based solution.

During the quenching process, the austenite phase ($\gamma^{\prime }$) is decomposed by a diffusionless transformation and complex shear mechanisms into small parallel arrays or stacks of lath-shaped martensitic crystals $\left(\alpha^{\prime }\right)$. The resulting lath microstructural characteristic is depicted in etched conditions in Figure 1a, by means of LOM. The peculiarity of a lath martensitic microstructure is its multi-level hierarchical nature: martensite lath, block and packet. Figure 1b elucidates the hierarchical morphology of the considered microstructure, by means of an etched section where PAG boundaries, packets and blocks become visible. The PAGs do not show a homogeneous grain size rather they show very heterogeneous grain sizes. The average PAG size was calculated with the line intersection method to approximately 12 μm. This result correlates well with the data from Koschella and Krupp [30].

**Figure 2:**
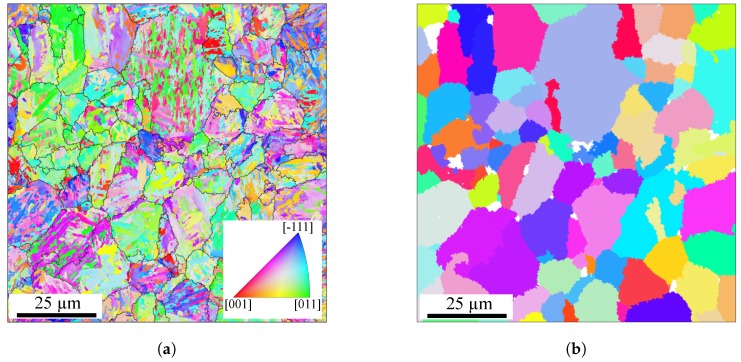
Martensitic microstructure of the SAE 4150. (**a**) EBSD map in inverse pole figure colour code with superimposed prior austenite grain boundaries. (**b**) Corresponding prior austenite grains calculated with ARPGE under assumption of Nishiyama-Wassermann orientation relationship.

EBSD analyses were performed on mechanically and electrically polished specimen in the SEM and areas of approximately 100 μm × 100 μm were scanned with a constant sampling step size of 0.10 μm to capture the microstructural variety in detail. Figure 2a shows the EBSD map in inverse pole figure colour code which clearly reveals the characteristic morphology of the lath martensitic microstructure in detail. According to the stereographic triangle in Figure 2a, the colours of the inverse pole figure correspond to the crystallographic directions normal to the investigated plane. This representation makes clear that the martensitic microstructure consists of multiple almost parallel crystallographic blocks forming morphological packets. The corresponding habit plane of these morphological packets are the family of the {111}${}_{\gamma^{\prime }}$ crystallographic planes.

The martensitic transformation of the investigated material can be well described by the Nishiyama-Wassermann orientation relationship (NW-OR) [31]. Based on the EBSD map of Figure 2a, the corresponding PAGs can be reconstructed with the software package ARPGE (Version: 1.6) [32] and the NW-OR. The corresponding PAG map is shown in Figure 2b, whereby the colour of a PAG indicates its crystallographic orientation. The white sections in Figure 2b indicate unsuccessful reconstructions of areas by ARPGE. Potential root causes therefore can be non-indexed pixels in the EBSD map, martensitic variants being far from the NW-OR, martensite laths belonging to two PAGs and for which it was impossible to decide [32].

### 2.2. Experimental Fatigue Properties

**Figure 3:**
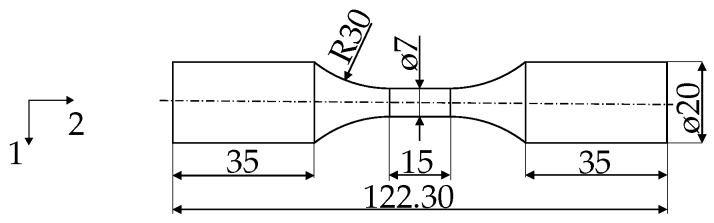
Geometry and dimensions (in mm) of the unnotched fatigue test specimens.

The fatigue crack initiation behaviour of the considered material was investigated by uniaxial strain-controlled fatigue tests which were performed according to the standards from ASTM E606 [33]. The fatigue experiments serving as reference for the micromechanical simulations were carried out at room temperature on a universal servo hydraulic test frame from MTS with 250 kN load capacity. In order to precisely measure the axial displacements in the gauge section, an extensometer from Sandner was mounted in the central zone of the unnotched fatigue specimen which is shown in Figure 3. The specimens have a precision-turned sample surface.

In total strain-controlled fatigue tests of smooth specimens, the cycles for macroscopic fatigue crack initiation are usually defined by a specific load drop of the maximum load [33,34]. In this study, the number of cycles at 10% load drop are correlated with macroscopic fatigue crack initiation [35]. However, this initial load drop is already associated with multiple cracks and corresponding crack lengths [36]. Due the experimental limitations in the determination of the required cycles for microscopic fatigue crack nucleation, the cycles of 10% load drop are linked to fatigue crack nucleation in this investigation. A triangular waveform with a constant strain rate of 0.02 s${}^{-1}$ was applied until the final fracture criterion of 50% load drop was reached, according to ASTM E606 [33].

**Figure 4:**
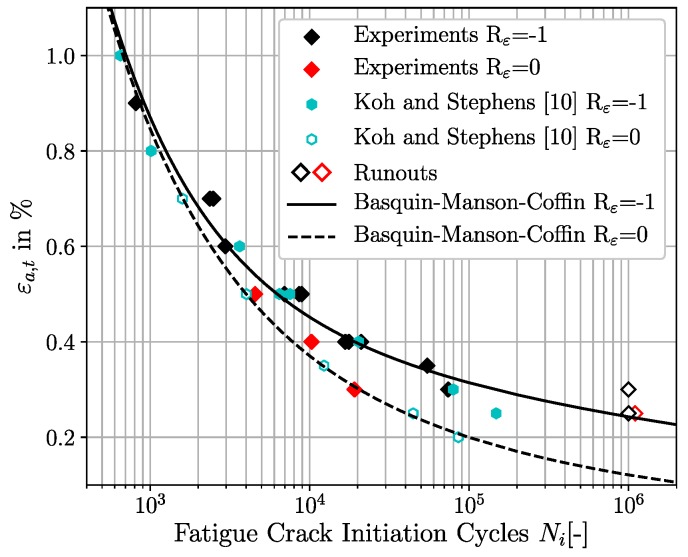
Experimental strain-controlled fatigue crack initiation test results of unnotched SAE 4150 (39 HRC) and ASTM A723 grade 1 (40 HRC) [10] at two different total strain ratios, R${}_{\varepsilon }$ = −1 and R${}_{\varepsilon }$ = 0.

Fully reversed strain-controlled fatigue experiments at R${}_{\varepsilon }$ = −1 were performed for total strain amplitudes ranging from $\varepsilon_{a,t}\in$ [0.25%; 0.90%]. Further four experiments with superimposed mean strains at R${}_{\varepsilon }$ = 0 were carried out for total strain amplitudes of 0.25%, 0.30%, 0.40% and 0.50%. In order to increase the experimental fatigue crack initiation database for R${}_{\varepsilon }$ = 0, fatigue crack initiation data of a comparable martensitic steel ASTM A723 grade 1 with 40 HRC from Koh and Stephens [10] are also taken into account. Beside the microstructural similarity of both materials, there is also a sufficient agreement of the monotonic mechanical properties between the SAE 4150 and the ASTM A723 grade 1. The monotonic properties of the ASTM A723 grade 1 are a Young’s modulus E = 200 GPa, a surface hardness of 40 HRC, a yield strength $\sigma_{f}=1170$ MPa, an ultimate tensile strength $\sigma_{u}=1262$ MPa and an elongation of 13.0%. A linear correlation plot of the fatigue crack initiation cycles at R${}_{\varepsilon }$ = −1 between the SAE 4150 and the ASTM A723 grade 1 exhibits an excellent agreement of both materials with respect to fatigue crack initiation behaviour between $\varepsilon_{a,t}=1.50\%$ and $\varepsilon_{a,t}=0.30\%$. This enables the use of the fatigue crack initiation data of the ASTM A723 grade 1 at R${}_{\varepsilon }$ = 0 as a reference for the subsequent simulations. The macroscopic experimental fatigue crack initiation lifetime results for both strain ratios are shown in Figure 4 by different coloured markers, where the total strain amplitude $\varepsilon_{a,t}$ is plotted versus the fatigue crack initiation cycles $N_{i}$. This visualisation shows the good correlation of the fatigue crack initiation data at R${}_{\varepsilon }$ = 0 between the ASTM A723 grade 1 and the SAE 4150 (red diamonds and cyan framed circles).

According to Figure 4, the effect of an increased strain ratio becomes apparent for only small total strain amplitudes where the positive mean stress reduces fatigue life significantly. On the other hand, for large applied total strain amplitudes, the fatigue life is almost independent of the strain ratio. This characteristic can be traced back to the mean stress relaxation behaviour of the considered material. An investigation from Wehner and Fatemi [11] on a comparable martensitic steel shows a good correlation with the presented experimental fatigue results of this study. For the sake of completeness, a detailed overview of the cyclic behaviour and mean stress relaxation characteristic of the investigated SAE 4150 is given in Schäfer et al. [31].

The experimental macroscopic fatigue crack initiation lives of the SAE 4150 for R${}_{\varepsilon }$ = −1 and of the ASTM A723 grade 1 for R${}_{\varepsilon }$ = 0 are represented in Figure 4 as solid black and dashed black curves by the four parametric strain life approach [4,33], respectively. This approach is a combination of a reformulated Basquin law [37] and the Manson-Coffin relationship [38,39,40] and can be expressed by the superposition of the elastic and plastic strain amplitudes [4,15,33]:

(1) $$\varepsilon_{a,t}=\varepsilon_{a,el}+\varepsilon_{a,pl}=\frac{\sigma_{f}^{{}^{\prime }}}{E}{\left(2N_{i}\right)}^{b}+\varepsilon_{f}^{{}^{\prime }}{\left(2N_{i}\right)}^{c}$$

where $\varepsilon_{a,t}$, $\varepsilon_{a,el}$ and $\varepsilon_{a,pl}$ are the total strain amplitude, elastic strain amplitude and plastic strain amplitude, respectively. $2N_{i}$ is the number of cycles for crack initiation; $\sigma_{f}^{{}^{\prime }}$ is the fatigue strength coefficient; *E* is the Young’s modulus; $\varepsilon_{f}^{{}^{\prime }}$ is the fatigue ductility coefficient and *b* and *c* represent the fatigue strength and fatigue ductility exponent, respectively. For the derivation of the mentioned coefficients and exponents of the presented law in Equation (1), the experimental fatigue data are separated into elastic and plastic strain amplitudes with a subsequent simple power law curve fitting procedure.

The superposition of elastic and plastic components in Equation (1) enables the description of the fatigue crack initiation resistance in terms of the total strain amplitude. In this context, the Basquin law principally addresses the HCF-regime with the elastic strain amplitudes and the Manson-Coffin relationship the LCF-regime with the plastic strain amplitudes [15]. It is worth noting, that the fatigue crack initiation curves according to Equation (1) without horizontal fatigue limits represent conservative estimations of fatigue crack initiation lives for the low strain amplitudes. Table 2 summarizes the parameters for the four parametric fatigue crack initiation cuves of Equation (1) for SAE 4150 for R${}_{\varepsilon }$ = −1 and of the ASTM A723 grade 1 for R${}_{\varepsilon }$ = 0. The power law curve fitting procedure for the coefficients and exponents of Equation (1) of the test series of R${}_{\varepsilon }$ = −1 and R${}_{\varepsilon }$ = 0 were performed independently of each other.

## 3. Modelling Methodology and Constitutive Model

The objective of the present study is the FCI prediction for SAE 4150 under different strain-controlled cycling loading conditions. For this, a micromechanical model is used enabling the calculation of the evolution of inhomogeneous stress and strain fields within the microstructures being characteristic for fatigue loading. This section deals with the description of the individual contributors to the micromechanical model, including in Section 3.1 the microstructure generation, in Section 3.2 the constitutive model, in Section 3.3 the local fatigue indicator parameters, in Section 3.4 the non-local homogenisation method and in Section 3.5 a fatigue crack initiation model.

### 3.1. Martensitic Microstructure Generation

Martensitic representative volume elements were generated by multiscale Voronoi tessellations within a framework of the C++ software library voro++ (Version: 0.4.6) and python (Version: 2.7). This martensitic microstructure generator has been developed in a previous study by the authors [31] and has proven to be adequate for micromechanical simulations of the considered material.

According to McDowell and Dunne [16] and Przybyla et al. [41], it is not sufficient to select a single numerical instantiation of the considered microstructure if fatigue crack initiation is the relevant response of interest. Rather, it is required to build up statistics based on multiple computational instantiations. Thus, the different microstructural realisations become statistical volume elements for the locally varying heterogeneous response. An RVE distinguishes itself from an SVE, that the response of the RVE will be location independent of a microstructural composition (e.g., effective cyclic stress-strain hysteresis) [42].

**Figure 5:**
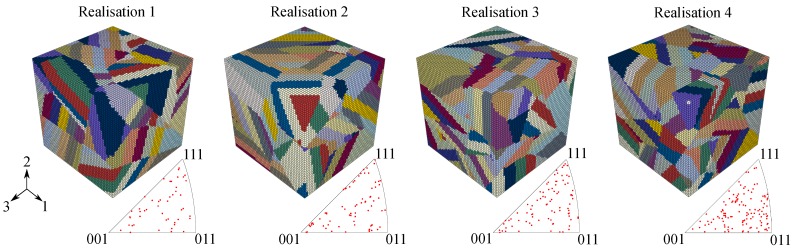
Representation of the four used martensitic statistical volume elements, including the corresponding pole figures characterising the crystallographic features of each realisation.

In order to account for the microstructural influence on fatigue crack initiation, four SVEs were generated in the present study. These microstructural instantiations which are depicted in Figure 5 incorporate the NW-OR for the appropriate crystallographic representation of lath martensite in SAE 4150 [31]. The realisations differ with respect to crystallographic properties, number of PAGs, number of crystallographic packets and number of blocks. Furthermore, random orientations were assigned to the PAGs. Table 3 shows the corresponding properties of the different realisations. Each SVE consist of 50 × 50 × 50 = 125,000 eight-node hexahedral linear brick elements with full integration (C3D8 in Abaqus).

Periodic boundary conditions are applied on opposite faces of the SVEs in order to simulate bulk material behaviour based on the work of Smit et al. [43]. In the current stage of model development, free specimen surfaces are not taken into account. The SVEs are subjected to axial cyclic strain-controlled loading in direction 2 with constant total strain amplitudes. Cyclic displacements are assigned to the master node so that the deformation behaviour of two opposite faces perpendicular to loading axis is controlled. The remaining four surfaces parallel to the loading axes are only constrained by the definition of the periodicity. The micromechanical simulations were performed with an applied strain rate of 0.009 s${}^{-1}$.

The different SVEs were simulated at two different total strain ratios, at R${}_{\varepsilon }$ = −1 and R${}_{\varepsilon }$ = 0. According to Castelluccio [44], saturated micromechanical fields arise typically after three to ten simulations cycles for fully reversed loading conditions. However, Manonukul and Dunne [25] postulate that the stabilisation process takes less than two cycles. In order to account for these fundamental results, the fully reversed simulations at R${}_{\varepsilon }$ = −1 were simulated for four cycles, in the present study. However, in order to account for mean stress relaxation effects at loading conditions with superimposed mean strains, the simulations at R${}_{\varepsilon }$ = 0 were performed for 15 simulation cycles. Accordingly, the FIPs can also be considered as fully stabilised for the increased strain ratio. For R${}_{\varepsilon }$ = −1, simulations were performed for nine different total strain amplitudes ranging from $\varepsilon_{a,t}$ = 0.225% to $\varepsilon_{a,t}$ = 0.90%. Due to the computational expense of the simulations at R${}_{\varepsilon }$ = 0, only seven selected characteristic strain levels were simulated ranging from $\varepsilon_{a,t}$ = 0.225% to $\varepsilon_{a,t}$ = 0.75%. All simulations were performed with 24 CPUs in parallel. Table 4 references the underlying simulation environment and the computational effort required to perform one micromechanical simulation with the given setup.

### 3.2. Constitutive Model

The micromechanical model used in the present study consists of a local phenomenological crystal plasticity (CP) model incorporated into the finite element solver Abaqus 2016 in form of a user material subroutine (UMAT). In a previous study by the authors, the size independent CP model was adapted and extended by kinematic hardening models for the simulation of the cyclic deformation behaviour of the investigated material. The model is able to capture adequately the Bauschinger effect as well as the mean stress relaxation behaviour of SAE 4150 for different strain ranges and strain ratios. The calibration, implementation and model description is described in detail in [31]; however the key equations are outlined here for the purpose of completeness.

The local CP model is implemented in a finite strain framework where the total deformation gradient F can be multiplicatively decomposed into an elastic stretching $F^{e}$ and plastic deformation $F^{p}$ component

(2) $$F=F^{e}F^{p}.$$

Plastic deformation in body-centred cubic (BCC) martensite may include in-lath- and out-of-lath-plane slip systems, the {110}<111> and {112}<111>, respectively. According to Michiuchi et al. [45], the {110} in-lath-plane slip systems are preferentially activated. Thus, only the twelve {110}<111> slip systems are evaluated in the present study. Thereby, the plastic velocity gradient $L^{p}$ can be expressed as the superposition of the shear rates of the different slip systems α by

(3) $$L^{p}={\dot{F}}^{p}{F^{p}}^{-1}=\sum_{\alpha =1}^{12}{\dot{\gamma }}^{\alpha }m^{\alpha }⊗n^{\alpha }$$

where ${\dot{\gamma }}^{\alpha }$, $m^{\alpha }$ and $n^{\alpha }$ are the shear rate, the unit vector in slip direction and the unit vector normal to the slip plane of the slip system α, respectively [46]. The phenomenological visco-plastic flow rule, for the shear rate ${\dot{\gamma }}^{\alpha }$, is formulated as a function of the resolved shear stress $\tau^{\alpha }$ and the critical resolved shear stress $\tau_{c,0}^{\alpha }$ as suggested by Rice, Hutchinson and Pierce et al. [46,47,48]

(4) $${\dot{\gamma }}^{\alpha }=\dot{\gamma_{0}}{\left|\frac{\tau^{\alpha }-\chi_{b}^{\alpha }}{\tau_{c,0}^{\alpha }}\right|}^{m}sign\left(\tau^{\alpha }-\chi_{b}^{\alpha }\right)\;$$

where $\dot{\gamma_{0}}$, $\tau^{\alpha }$, $\tau_{c,0}^{\alpha }$ and $\chi_{b}^{\alpha }$ are the reference shear rate, the resolved shear stress, the critical resolved shear stress and the back stress of the slip system α, respectively. The exponent of the power law is represented by *m*. Since the modelling of cyclic material behaviour requires the consideration of kinematic hardening, a back stress variable $\chi_{b}^{\alpha }$ is incorporated in the flow rule formulation. In the present study, a modified version of the Ohno Wang [49] kinematic hardening model is used. Thus, the evolution of the back stress ${\dot{\chi }}_{b}^{\alpha }$ on the crystal level is given by

(5) $${\dot{\chi }}_{b}^{\alpha }=A{\dot{\gamma }}^{\alpha }-B{\left(\frac{|\chi_{b}^{\alpha }|}{\frac{A}{B}}\right)}^{M}\chi_{b}^{\alpha }\left|{\dot{\gamma }}^{\alpha }\right|$$

where *A*, *B* and *M* are material specific constants [50].

**Figure 6:**
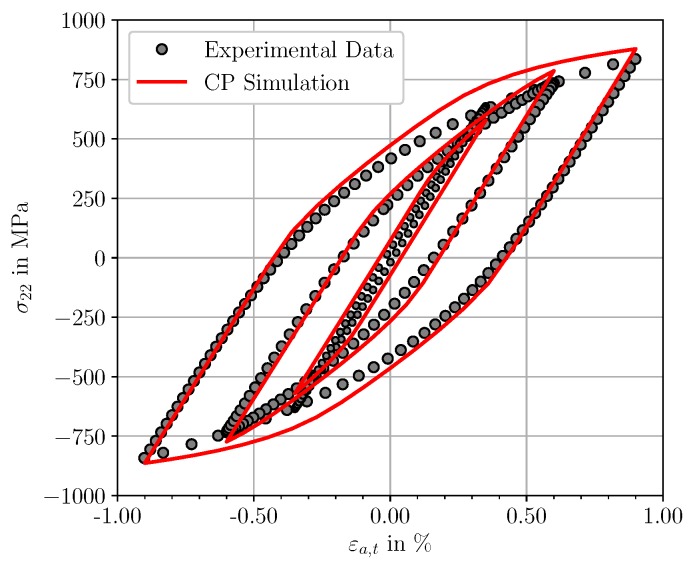
Stabilised cyclic stress-strain hystereses extracted at half-life of the LCF-experiments (grey markers) and the calibrated hystereses using the proposed crystal plasticity model and the corresponding parameter set (red curves).

The parameters of the local CP model for fatigue simulation of the material are presented in Table 5. The elastic constants reported by Xie et al. [51] show a good agreement between experimental data and simulation data within the elastic regime. Values for the reference shear rate ${\dot{\gamma }}_{0}$ and the strain rate sensitivity exponent are taken from literature [28,52]. The remaining parameters of the CP model were calibrated to experimental stress-strain data of the considered SAE 4150 in [31] using an multi-objective calibration procedure. Figure 6 shows three different experimental cyclic stress-strain hystereses as well as the corresponding simulated cyclic stress-strain hystereses with a qualitative good agreement. However, a quantitative comparison of the plastic strain energy densities (hystereses areas) between the experimental and simulated stress strain hystereses indicates deviations of 0.39 MJ/m${}^{3}$ (157%), 0.009 MJ/m${}^{3}$ (0.3%), and 0.67 MJ/m${}^{3}$ (6.8%) for the total strain amplitudes of 0.35%, 0.60% and 0.90%, respectively. The increased level of plastic strain energy density at $\varepsilon_{a,t}=0.35\%$ is caused by the numerical overestimation of the plasticity levels for small applied strain amplitudes with the given set of crystal plasticity parameters. A comprehensive discussion of the plastic strain energy density characteristics between the experimentally observed values and the micromechanical predicted values is given in Schäfer et al. [31].

### 3.3. Local Fatigue Indicator Parameters

Microstructure-sensitive modelling environments use surrogate measures to link micromechanical quantities with fatigue damage. These surrogate measures are mesoscopic fatigue indicator parameters representing driving forces for fatigue crack nucleation and microstructurally short crack growth [16]. FIPs provide microstructure depending information about the evolution of stress and strain fields during a fatigue loading cycle. The fundamental assumption of FIPs is that irreversible slip on the glide systems contribute to fatigue damage. In the present study, three different FIP formulations have been selected from literature (i.e., [16,25,53]), to assess the correlation between predicted and experimentally observed fatigue crack initiation.

First, the accumulated plastic slip FIP${}_{p}$ has been reported by Manonukul and Dunne [25] as a fundamental quantity for fatigue crack initiation. The dimensionless scalar quantity reflects the complete plastic deformation history of a material point. Under cycling loading conditions, the accumulated plastic slip increases monotonically due to classical to-and-fro slip of dislocations. The FIP${}_{p}$ can be calculated by a double contraction of the plastic velocity gradient $L_{p}$ and a subsequent time integration:

(6) $${\mathrm{FIP}}_{p}=\int_{0}^{t}\dot{p}\;dt=\int_{0}^{t}\sqrt{\frac{2}{3}L_{p}:L_{p}}\;dt$$

The second FIP investigated in this study is represented by the Fatemi-Socie parameter ${\mathrm{FIP}}_{\mathrm{FS}}$ [24]. This shear-based criterion has been introduced to micromechanical modelling by McDowell [54]. A number of investigations have shown that the ${\mathrm{FIP}}_{\mathrm{FS}}$ correlate well with multiaxial fatigue crack initiation data in both LCF- and HCF- regime. At the crystal scale, the ${\mathrm{FIP}}_{\mathrm{FS}}$ considers the combination of cyclic plastic slip and the corresponding normal stress of the slip plane. The dimensionless scalar quantity can be expressed by:

(7) $${\mathrm{FIP}}_{\mathrm{FS}}=max{\left[\frac{\Delta \gamma_{p}^{\alpha }}{2}\left(1+k\frac{\sigma_{n}^{\alpha }}{\tau_{c,0}}\right)\right]}_{\alpha =1...12}$$

where $\Delta \gamma_{p}^{\alpha }$ is the local plastic shear strain range of the cycle at each slip system, $\sigma_{n}^{\alpha }$ is the corresponding maximum normal stress to the plane of $\Delta \gamma_{p}^{\alpha }$, $\tau_{c,0}$ is the initial critical resolved shear stress and *k* represents a constant which controls the influence of the normal stress.

The third FIP used in this study assumes fatigue crack initiation to be associated with energy dissipation due to plastic slip on the slip planes. Skelton [55] suggested that this metric could be associated with the energy required to propagate a fatigue crack. In the context of micromechanical modelling, Korunsky et al. [53] proposed an energy dissipation criterion that considers the energy dissipated over all slip systems. The criterion is defined within the given crystallographic context by

(8) $${\mathrm{FIP}}_{W}=\sum_{\alpha =1}^{12}\int_{0}^{t}\tau^{\alpha }{\dot{\gamma }}^{\alpha }\;dt$$

where $\tau^{\alpha }$ and ${\dot{\gamma }}^{\alpha }$ are the resolved shear stress and the shear rate of the slip system α, respectively.

### 3.4. Non-Local Averaging

The FIPs presented in Section 3.3 are calculated by the CP model in the centre of each finite element of the SVE. These local quantities dependent strongly on the details of the discretization of the SVE and do not account for the material specific finite volume of the fatigue damage process zone [56]. This can be justified by the fact that crack embryos and dislocation substructures form in the order of several sub-micrometers or in the low μm-range. In order to avoid mesh sensitivity and to account for the finite fatigue damage zone, the FIPs should be homogenized over a region representative of the material specific crack incubation zone [16,56]. Castelluccio [44] introduced such post-processing regularization schemes in form of a sphere averaging as well as a band averaging techniques.

Experimental investigations from Du et al. [7] on lath martensite show that block boundaries act as an effective barrier for dislocation movement. In contrast, sub-block boundaries can be crossed by dislocations. Hence, a sphere-averaging homogenisation method for the local FIPs within the individual martensitic blocks is proposed in the present study. The homogenisation technique for the different non-local fatigue indicator parameter, ${\mathrm{FIP}}_{i}^{*}$, can be defined by

(9) $${\mathrm{FIP}}_{i}^{*}=\frac{1}{V_{pz}}\int_{V_{pz}}{\mathrm{FIP}}_{i}\;dV$$

where $V_{pz}$ represents the process zone volume for fatigue crack initiation and ${\mathrm{FIP}}_{i}$ the selected FIP, whereby the process zone volume is not allowed to cross the block boundary. The volume averaged data are reassigned to the considered integration points, after homogenisation. According to Prithivirajan and Sangid [57], the spatially restricted homogenisation within individual grains and blocks enables the preservation of gradients across grain boundaries due to the kinematic compatibility and the elastic anisotropy. A weighting of the data points within the averaging sphere is neglected in the proposed homogenisation scheme due to the limited spatial extension of the martensitic blocks. The validity of the implementation was proven by the Hill-Mandel [58] condition assuming equality of the virtual work from macro- and micro-scale.

### 3.5. Fatigue Crack Initiation Model

According to Manonukul and Dunne [25], fatigue crack nucleation occurs once a critical value of the accumulated plastic slip is reached on the micro-level. Numerous investigations have shown that this hypothesis can be extended from the accumulated plastic slip to the class of several FIPs, as for example the Fatemi-Socie parameter and the dissipated energy [16,23,59].

The material specific critical value of the corresponding fatigue indicator parameter, ${FIP}_{crit}$, can be determined by a LCF-experiment at a defined total strain amplitude $\varepsilon_{a,t}^{†}$ with the fatigue crack initiation lifetime $N_{i}^{†}$ and a corresponding micromechanical simulation. The corresponding simulation is performed at equal loading conditions with the total strain amplitude $\varepsilon_{a,t}^{†}$ until saturation of the FIPs is reached. In analogy to Manonukul and Dunne [25], the critical value of the corresponding fatigue indicator parameter ${FIP}_{crit}$ can then be calculated by

(10) $${FIP}_{crit}=N_{i}^{†}\cdot \Delta {FIP}_{cyc}^{*}\left(\varepsilon_{a,t}^{†}\right)$$

where $N_{i}^{†}$ and $\Delta {FIP}_{cyc}^{*}\left(\varepsilon_{a,t}^{†}\right)$ represent the experimentally observed fatigue crack initiation lifetime and the non-local saturated cyclic FIP at corresponding loading conditions, respectively. Under the assumption that the ${FIP}_{crit}$ is independent of the loading level and the total strain ratio, a linear fatigue life model can then be expressed by

(11) $$N_{crit}=\frac{{FIP}_{crit}}{\Delta {FIP}_{cyc}^{*}}$$

where $N_{crit}$ represents a the critical number of cycles to initiate a microcrack and $\Delta {FIP}_{cyc}^{*}$ the corresponding saturated FIP. It should be noted that the critical FIP values have been calibrated by using LCF data. In this study, the critical values are also used for fatigue crack initiation predictions in the HCF regime, based on the hypothesis that the critical FIP keeps constants across the LCF as well as the HCF regime, as demonstrated by Manonukul and Dunne [25]. The proposed methodology enables the creation of fatigue crack initiation life time diagrams as a function of microstructure morphology, hardening state and defect distribution [21]. In the present study, the stated criterion in Equation (11) is used for the determination of the early stage of fatigue crack initiation, the so called fatigue crack nucleation. The subsequent fatigue stages like growth of MSC and PSC can also be modelled with the proposed methodology in general and will be object of future investigations.

## 4. Results and Discussion

The micromechanical model described in the previous section is used to investigate the early stage of the fatigue crack initiation behaviour of the considered material, in a fundamental way. First, the total strain amplitude dependent heterogeneities of stress and strain fields are evaluated. Next, comparisons of the three different non-local FIPs are performed for two considered strain ratios, R${}_{\varepsilon }$ = −1 and R${}_{\varepsilon }$ = 0. Finally, the results are compared and discussed in detail. Thereby, the micromechanical fatigue crack initiation life is compared with the experimental macroscopic fatigue crack initiation life.

### 4.1. Heterogeneity of Stress and Strain Fields

**Figure 7:**
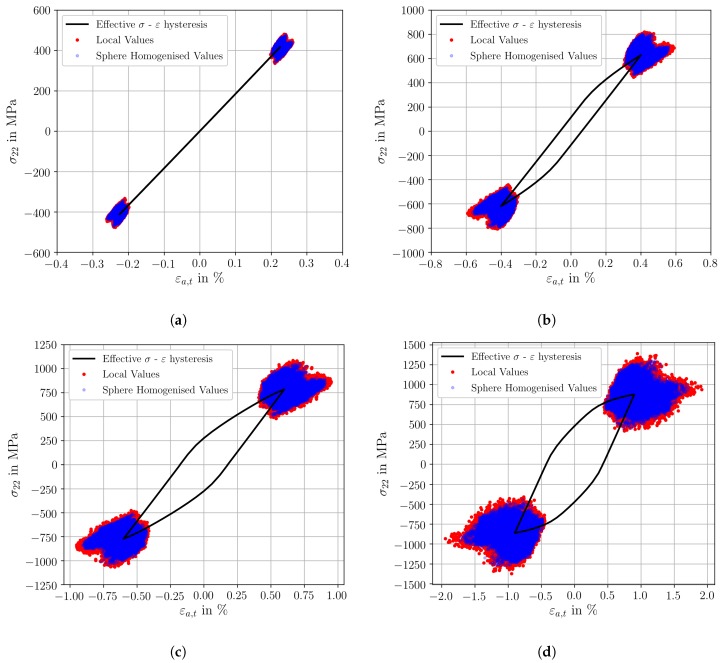
Effective cyclic stress-strain hystereses, local integration point values of stress and strain (red markers) and corresponding sphere homogenised values of stress and strain (blue markers), for (**a**) $\varepsilon_{a,t}=0.225\%$, (**b**) $\varepsilon_{a,t}=0.40\%$, (**c**) $\varepsilon_{a,t}=0.60\%$ and (**d**) $\varepsilon_{a,t}=0.90\%$.

In the following, four different micromechanical simulations at R${}_{\varepsilon }$ = −1 are evaluated for the prediction of the cyclic effective behaviour as well as for the corresponding local stress and strain heterogeneities. Here, loading conditions from the macroscopic purely elastic up to fully elastic plastic response are taken into account. The stabilised cyclic effective stress-strain hystereses of the SVE1 are shown as solid black lines for $\varepsilon_{a,t}$ = 0.225%, $\varepsilon_{a,t}$ = 0.40%, $\varepsilon_{a,t}$ = 0.60% and $\varepsilon_{a,t}$ = 0.90% in Figure 7a–d, respectively. In addition, local stress-strain tuples (i.e., $\sigma_{22}$ and $\varepsilon_{22}$ (Green-Lagrangian strains)) of the individual integration points are represented as red dots for the maximum and minimum applied total strain within a loading cycle. Furthermore, the corresponding sphere homogenised values are plotted as blue dots.

Considering Figure 7a–d, it can be observed, that the sphere homogenised values (blue dots) show a good agreement with the spatial distribution of the local integration point values (red dots), only with a lower order of magnitude due to the averaging procedure. According to Figure 7a, for $\varepsilon_{a,t}$ = 0.225% the effective cyclic stress-strain hysteresis represents a macroscopic elastic material behaviour. The corresponding sphere homogenised stress-strain tuples are rather homogeneous. For the maximum applied strain, the strains vary from 0.20% to 0.25% (Δε = 0.05%) and the stresses from 340 MPa to 470 MPa (Δσ = 130 MPa). On the contrary, increased inhomogeneous stress-strain tuples arise for macroscopically elastic-plastic loadings. Figure 7d shows the cyclic effective stress-strain hysteresis for $\varepsilon_{a,t}$ = 0.90% and the substantial scatter of the sphere homogenised stress-strain data. The sphere homogenised strains vary from 0.47% to 1.71% (Δε = 1.24%) and the sphere homogenised stresses from 449 MPa to 1305 MPa (Δσ = 856 MPa). The comparison of Figure 7a–d indicate that the scatter of sphere homogenised stress and strain heterogeneities increase with increasing total strain amplitude.

A comparison of the four loading conditions via the absolute strain scatter Δε is less suitable than a consideration of the fraction of the maximum sphere homogenised strain to the maximum strain of the σ-ε hysteresis for each applied total strain amplitude. The macroscopic elastic loading in Figure 7a result in a strain concentration factor of 1.14 times the homogenised applied strain, whereby the remaining loading conditions result in strain concentration factors of 1.39, 1.51 and 1.90 for $\varepsilon_{a,t}=0.40\%$, $\varepsilon_{a,t}=0.60\%$ and $\varepsilon_{a,t}=0.90\%$, respectively. Transferring this evaluation methodology to the sphere homogenised stresses, stress concentration factors arise of 1.14, 1.27, 1.34 and 1.48 for $\varepsilon_{a,t}=0.225\%$, $\varepsilon_{a,t}=0.40\%$, $\varepsilon_{a,t}=0.60\%$ and $\varepsilon_{a,t}=0.90\%$, respectively. Multiple experimental observations by high resolution digital image correlation on different crystal structures confirm the order of magnitude of the observed strain concentration factor for macroscopically elastic-plastic loadings [60,61]. An evaluation of the strain and stress concentration factors shows, that the heterogeneity of sphere homogenised strains and stresses increases linearly by increasing the total strain amplitude, whereby the scatter of the sphere homogenised strains increase most.

### 4.2. Comparison of Fatigue Indicator Parameters at R${}_{\varepsilon }$ = −1

The comparison of the three different FIPs from Section 3.3 requires the definition of the corresponding critical FIPs, in a first step. For this purpose, a micromechanical simulation was performed with SVE1 with a total strain amplitude of $\varepsilon_{a,t}=0.60\%$ at R${}_{\varepsilon }$ = −1. The choice of this total strain amplitude is justified both by the excellent agreement in the plastic strain energy density level with the experimentally observed values and due to fact that the total strain amplitude of $\varepsilon_{a,t}=0.60\%$ represents an intermediate level between the maximum and minimum applied total strain amplitude in the present study. Saturated local FIPs and sphere homogenised, termed as non-local FIPs, were extracted from the simulation results via post-processing steps. The critical FIPs were determined by using Equation (10), the extracted saturated simulated FIPs and the corresponding experimental number of cycles to FCI. For the sake of completeness, the experimental FCI time was extracted from the Basquin-Manson-Coffin representation of Equation (1) to obtain a statistical averaged value. Table 6 summarises the local and non-local critical FIPs for the considered material.

**Figure 8:**
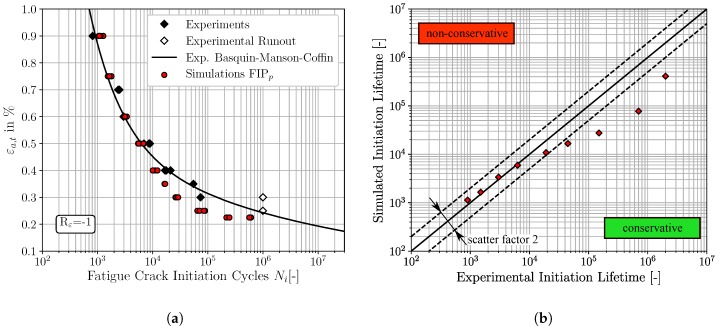
Comparisons of the fatigue crack initiation predictions based on the non-local accumulated plastic slip and the experimental fatigue crack initiation lifetime, for R${}_{\varepsilon }=-1$: (**a**) ε-N-curve (**b**) lifetime correlation plot.

**Figure 9:**
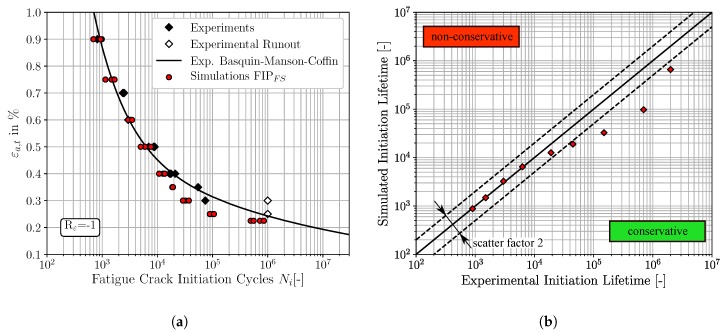
Comparisons of the fatigue crack initiation predictions based on the non-local Fatemi-Socie parameter and the experimental fatigue crack initiation lifetime, for R${}_{\varepsilon }=-1$: (**a**) ε-N-curve (**b**) lifetime correlation plot.

**Figure 10:**
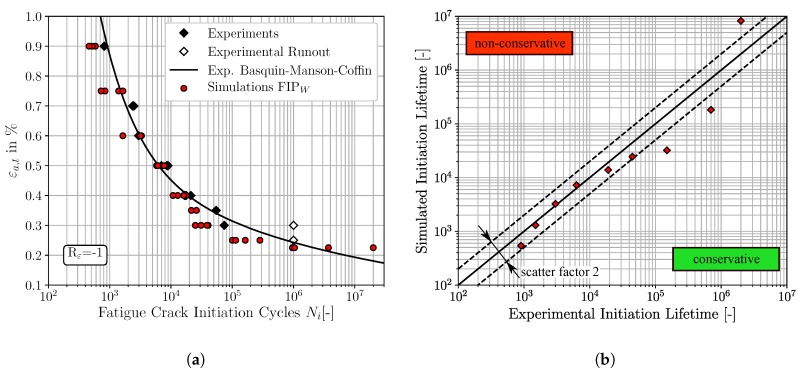
Comparisons of the fatigue crack initiation predictions based on the non-local energy dissipation criteria and the experimental fatigue crack initiation lifetime, for R${}_{\varepsilon }=-1$: (**a**) ε-N-curve (**b**) lifetime correlation plot.

The micromechanical FCI predictions for fully reversed loading conditions of the three different non-local FIPs, accumulated plastic slip, Fatemi-Socie and energy dissipation criteria, are depicted in Figure 8, Figure 9 and Figure 10, respectively. The result presentation is arranged as follows: Figure 8a, Figure 9a and Figure 10a represent the traditional ε-N-curves, whereby the experimental results are plotted as black diamonds, experimental runouts as black framed diamonds, the corresponding experimental Basquin-Manson-Coffin relation as solid black line and the simulated microstructure dependent results as red dots. On the other hand, the Figure 8b, Figure 9b and Figure 10b represent lifetime correlation plots whereby the arithmetic averaged simulated FCI cycles are plotted against the corresponding experimental FCI cycles. In this way of representation, the solid black line under 45° represents the condition of perfect agreement between experimental and simulated cycles for FCI. Following the way of representation of Lazzarin and Susmel [62] scatter bands of a factor of two are also shown as dashed black lines corresponding to the half or the twice of the considered lifetime. The experimental strain amplitude dependent cycles for FCI were extracted from the underlying Basquin-Manson-Coffin relation due to the limited number of available experimentally tested total strain amplitudes.

According to Figure 8a, the predicted cycles for FCI based on the non-local accumulated plastic slip show in general a good qualitative agreement with the experimental results. However, at the largest applied total strain amplitude, $\varepsilon_{a,t}=0.90\%$, the FCI cycles result in slightly non-conservative predictions, which are still within the scatter band of factor two. The four FCI predictions for total strain amplitudes smaller than $\varepsilon_{a,t}=0.40\%$ are located outside the scatter band, visualised in Figure 8b, and reveal increased deviations between the predicted and experimental observed FCI cycles.

The predictions of the FCI cycles based on the non-local Fatemi-Socie parameter exhibit also a good qualitative agreement to the experimental LCF-data, shown in Figure 9a. These FCI predictions are either located on or below the 45° correlation line in Figure 9b, implying that the results reveal an accurate but also a conservative character. Equivalent to the FCI predictions based on the FIP${}_{p}$, the FCI predictions based on the Fatemi-Socie parameter show also increased deviations to the experimental results for strain amplitudes smaller than $\varepsilon_{a,t}=0.40\%$. Furthermore, the different microstructural realisations show only small variations of the FCI cycles at constant strain amplitudes. The four simulated SVEs are of course only partially capable to reproduce scatter.

Finally, the qualitative comparison of the FCI predictions based on the non-local energy dissipation criteria and the experimental results show also a good agreement, Figure 10. Analogous to the Fatemi-Socie based predictions, the FIP${}_{W}$ predictions are either located close to or below the 45° correlation line. Thus, the prediction with this FIP reveals an accurate and conservative character, except for the smallest total strain amplitude of $\varepsilon_{a,t}=0.225\%$. Furthermore, the simulation results show an increased scatter for small total strain amplitudes in comparison to the predictions based on the FIP${}_{p}$ and the FIP${}_{FS}$. The observation from Sweeney et al. [23], that micromechanical predictions of cycles for FCI based on the FIP${}_{W}$ are in general marginally higher than based on FIP${}_{p}$, can be confirmed. Furthermore, the increase in scatter for cycles required for FCI can be attributed to the increased sensitivity to local stresses of the energy dissipation criteria [23].

All three simulations reveal increasing deviations to the experimental fatigue crack initiation lifetimes for total strain amplitudes below $\varepsilon_{a,t}=0.40\%$. As already mentioned in Section 3.2, the micromechanical simulation model tends to overestimate the level of plasticity for simulations at small strain amplitudes. According to Ellyin [4], increased levels of dissipation energy per cycle result in a reduction of fatigue life. Thus, the increased level of plasticity for small strain amplitudes may cause a too conservative estimation of the fatigue crack initiation life at R${}_{\varepsilon }$ = −1.

A second possibility for the increased deviations at small strain amplitudes could be the neglect of the microstructural short crack growth, in the present study. In the LCF-regime, the entire microstructure is extensively plastically deformed and the corresponding driving forces for crack growth are high to propagate the nucleated cracks up to the critical length. However, in the HCF regime with highly heterogeneous plastic strain localisations, the driving forces for MSC growth decline and also crack arrest become important for the total fatigue life [54]. According to Polák et al. [18], the lifetime in the HCF-regime is dominated by the cycles for crack nucleation as well as the cycles required to propagate the microstructurally small fatigue cracks.

### 4.3. Comparison of Fatigue Indicator Parameters at R${}_{\varepsilon }$ = 0

In the next step, the applicability of the different fatigue indicator parameters is assessed for a totally different strain ratio of R${}_{\varepsilon }=0$. To characterize the effect of an increased total strain ratio with respect to FCI cycles, micromechanical simulations with the four martensitic SVEs of Figure 5 were performed for 15 loading cycles at a strain ratio of R${}_{\varepsilon }=0$ with the equivalent numerical settings as in the previous section. The increased number of simulation cycles are needed to account for mean stress relaxation effects and consequently for a stabilisation of the FIPs. After 15 simulation cycles at R${}_{\varepsilon }=0$, the local as well as the non-local FIPs vary less than 0.5% and can be considered as fully stabilised cyclic FIPs.

**Figure 11:**
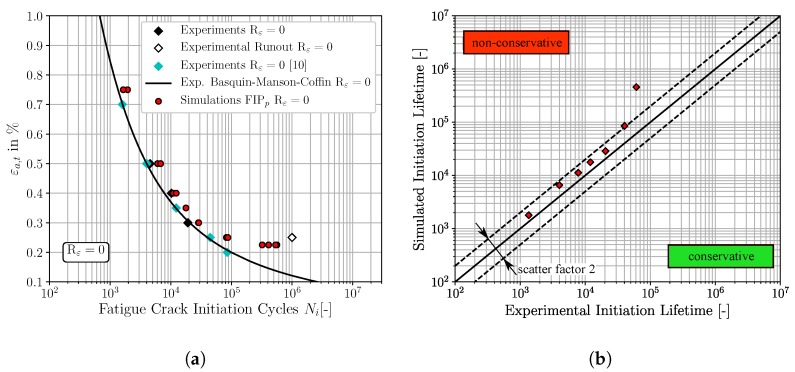
Comparisons of the fatigue crack initiation predictions based on the non-local accumulated plastic slip and the experimental fatigue crack initiation lifetime, for R${}_{\varepsilon }=0$: (**a**) ε-N-curve (**b**) lifetime correlation plot.

**Figure 12:**
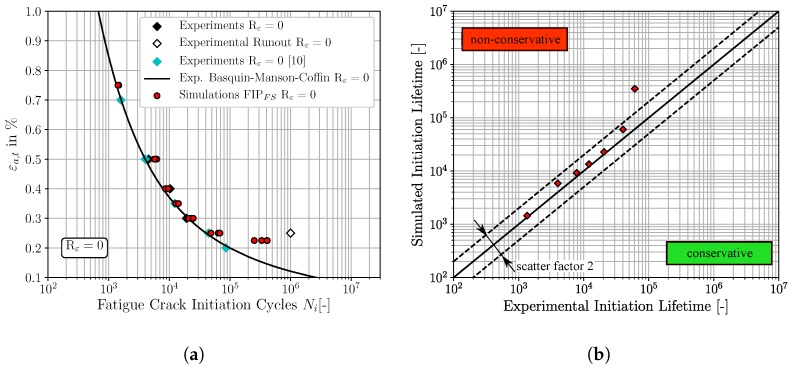
Comparisons of the fatigue crack initiation predictions based on the non-local Fatemi-Socie parameter and the experimental fatigue crack initiation lifetime, for R${}_{\varepsilon }=0$: (**a**) ε-N-curve (**b**) lifetime correlation plot.

**Figure 13:**
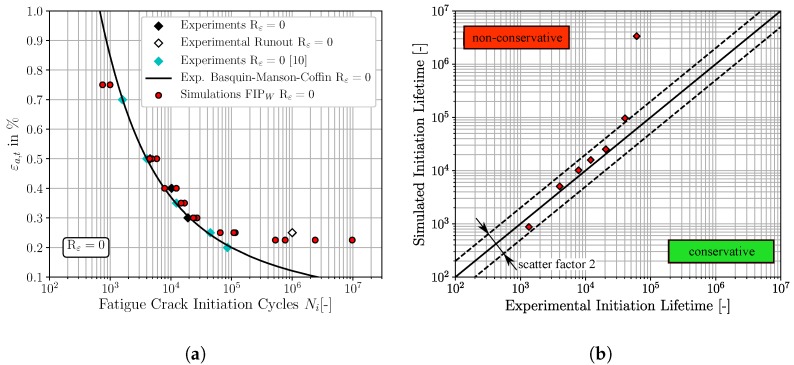
Comparisons of the fatigue crack initiation predictions based on the non-local energy dissipation criteria and the experimental fatigue crack initiation lifetime, for R${}_{\varepsilon }=0$: (**a**) ε-N-curve (**b**) lifetime correlation plot.

In analogy to the result representation of the previous Section 4.2, the Figure 11, Figure 12 and Figure 13 show the micromechanical FCI predictions for R${}_{\varepsilon }=0$ of the three different FIPs, accumulated plastic slip, Fatemi-Socie and energy dissipation criteria, respectively. Figure 11a, Figure 12a and Figure 13a represent the traditional ε-N-curves at R${}_{\varepsilon }=0$, whereby the experimental FCI results are plotted as black and cyan (results from Koh and Stephens [10]) diamonds, experimental runouts as black framed diamonds, the corresponding experimental Basquin-Manson-Coffin relation at R${}_{\varepsilon }=0$ as solid black line and the simulated microstructure dependent results as red dots. On the other hand, the Figure 11b, Figure 12b and Figure 13b represent lifetime correlation plots whereby the arithmetic averaged simulated FCI cycles at R${}_{\varepsilon }=0$ are plotted against the corresponding experimental FCI cycles at R${}_{\varepsilon }=0$.

According to Figure 11, the predictions of the required cycles for FCI at R${}_{\varepsilon }=0$ by the non-local accumulated plastic slip show a consistent non-conservative character due to the spatial distribution of the red markers above the 45° correlation line in Figure 11b. However, only the FCI cycles for $\varepsilon_{a,t}=0.225\%$ are located outside of the scatter band with a factor of 2. In contrast, the predictions of FCI at R${}_{\varepsilon }=0$ with the non-local Fatemi-Socie criterion are closer to the experimental Basquin-Manson-Coffin line and the 45° correlation line in Figure 12a,b, respectively. Consequently, the predictions with the non-local Fatemi-Socie criterion show a better agreement with the experimental FCI results than by means of the non-local accumulated plastic slip. Finally, the FCI predictions with the non-local energy dissipation criteria in Figure 13 show for the highest applied total strain amplitude a conservative character. With decreasing total strain amplitudes, the deviations to the experimental FCI results increase and the numerical predictions become non-conservative. Based on the results from Figure 11, Figure 12 and Figure 13 the FCI predictions by the non-local Fatemi-Socie criterion are superior to the FCI predictions by the accumulated plastic slip and energy dissipation criteria.

In addition to the result representation for R${}_{\varepsilon }=0$ via the traditional ε-N-curves and the FCI correlation plots, the effect of an increased strain ratio on the cycles required for FCI as well as the result consistency can also be evaluated by the comparison of the average cycles required for FCI at R${}_{\varepsilon }=-1$ and R${}_{\varepsilon }=0$. Therefore, the lifetime reduction factor Γ is defined by

(12) $$\Gamma =\frac{N_{i}\left(R_{\varepsilon }=-1\right)}{N_{i}\left(R_{\varepsilon }=0\right)}\;.$$

**Figure 14:**
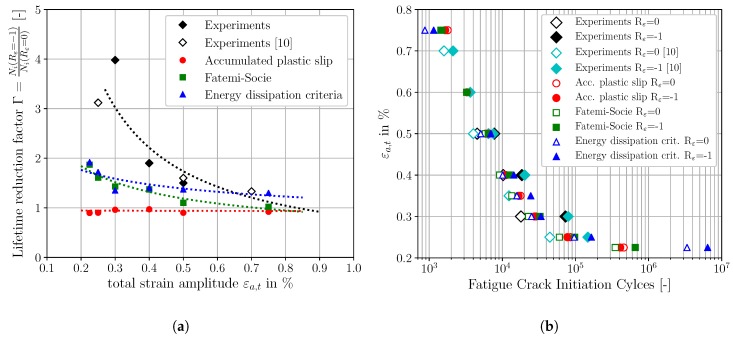
Evaluation of the effect of an increased strain ratio from R${}_{\varepsilon }=-1$ to R${}_{\varepsilon }=0$ on the average fatigue crack initiation behaviour: (**a**) Comparison of three different fatigue indicator parameters via a lifetime reduction factor. (**b**) Traditional ε-N plot of the experimental as well as simulated average fatigue crack initiation cycles.

An appropriate representation of the life time reduction factor is the visualisation of Γ vs. the applied total strain amplitude $\varepsilon_{a,t}$. Figure 14a shows the discrete lifetime reduction factors for the experimental as well as for the simulation results comprising the three different FIPs as solid markers. The simulations represent the average of the four simulated SVEs. As well, power regressions lines based on the corresponding life time reduction factors are plotted as dashed lines in the corresponding colours.

The experimental based lifetime reduction factors in Figure 14a indicate a smaller influence of strain ratio on FCI cycles for large applied total strain amplitudes. However, at lower total strain amplitudes the effect of an increased strain ratio becomes more evident by the reduction of FCI time up to a factor of 4.0. This characteristic behaviour can be explained by the phenomena of load-level dependent mean stress relaxation, which is more pronounced for large applied total strain amplitudes. Experimental results from Wehner and Fatemi [11] at a comparable martensitic steel confirm the observed characteristic. Since there is no information about the experimental scatter for the characteristic at R${}_{\varepsilon }=0$ and there are also augmented experimental results from literature used, only qualitative comparisons are made in the subsequent analysis based on Figure 14a.

The non-local accumulated plastic slip cannot capture the effect of an increased strain ratio. Regardless of the applied total strain amplitude, the lifetime reduction factor oscillates around Γ=1.0. At $\varepsilon_{a,t}=0.225\%$, $\varepsilon_{a,t}=0.25\%$ and $\varepsilon_{a,t}=0.35\%$ the FCI time at R${}_{\varepsilon }=0$ is even higher than at R${}_{\varepsilon }=-1$, indicating inconsistent results with the experimental observations. Consequently, the non-local accumulated plastic slip is not suited for FCI predictions for loading levels with superimposed mean strains, based on the present results. The lifetime reduction factor characteristic of the second investigated FIP, the Fatemi-Socie parameter, shows a qualitative good agreement with the corresponding experimental one. In principle, the experimentally observed characteristic can be modelled with increasing deviations at the lifetime reduction factors for small applied strain amplitudes. The root cause for these deviations is the increased level of plasticity for small strain simulations. Thereby, an increased level of mean stress relaxation occurs and the detrimental mean stresses decline resulting in an increased fatigue crack initiation lifetime for R${}_{\varepsilon }=0$. On the other hand, the increased level of plasticity at R${}_{\varepsilon }=-1$ results in reduced cycles for FCI due to the higher level of dissipated plastic energy and consequently the cycles for FCI of R${}_{\varepsilon }=-1$ and R${}_{\varepsilon }=0$ become more equivalent. Finally, the energy dissipation criteria shows also the possibility to predict correctly the influence of the increased strain ratio at small applied total strain amplitudes. However, the qualitative agreement with the experimentally observed characteristic of the lifetime reduction factor is less than the characteristic predicted by the non-local Fatemi-Socie criteria, in particular for large applied total strain amplitudes.

For the sake of completeness, the FIP dependent absolute average fatigue crack initiation cycles as well as the corresponding experimental values from Figure 14a are shown in Figure 14b in form of a traditional ε-N-plot. Based on this visualisation, the Fatemi-Socie and the energy dissipation criteria represent suited FIPs to perform FCI simulations under different strain ratios. Furthermore, the results of the Fatemi-Socie as well as the dissipated energy metric indicate a good transferability between the different applied total strain ratios and enable thereby predictions of FCI at different applied total strain ratios and strain amplitudes, in future.

### 4.4. Fatigue Crack Initiation Prediction of SAE 4150 for R${}_{\varepsilon }$ = −1 and R${}_{\varepsilon }$ = 0

**Figure 15:**
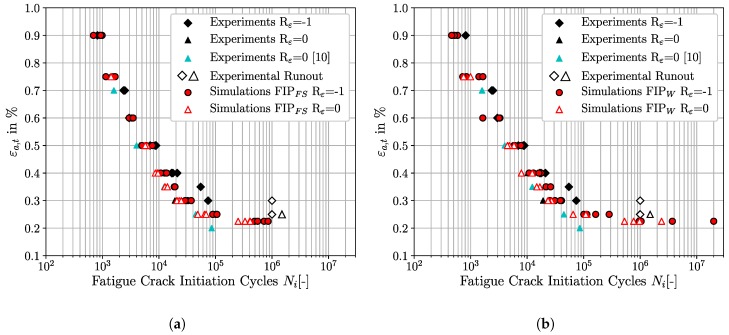
Experimental and simulated fatigue crack initiation lifetimes for $R_{\varepsilon }$ = −1 and R${}_{\varepsilon }$ = 0 for the investigated SAE 4150. (**a**) Micromechanical fatigue crack initiation predictions with the non-local Fatemi-Socie criteria. (**b**) Micromechanical fatigue crack initiation predictions with the non-local energy dissipation criteria.

Based on the previous results from Section 4.2 and Section 4.3 the non-local Fatemi-Socie metric as well as the dissipated energy criterion present the most suited criteria to predict FCI for the considered material for a variety of loading conditions. Figure 15a,b summarize the experimental as well as the simulated FCI results for R${}_{\varepsilon }=-1$ and R${}_{\varepsilon }=0$ in traditional ε-N-plots for the Fatemi-Socie metric and the dissipated energy criteria, respectively.

The characteristics of the fatigue crack initiation lifetime scatter can be pointed out by means of Figure 15a,b. In general, the scatter of FCI cycles at constant loading levels is caused by the different crystallographic and morphological realisations of the investigated SVEs. The increase in scatter from the LCF to HCF-regime is caused by the decline of total strain amplitude. The overall level of plasticity is thereby reduced and solely highly heterogeneous plastic strain localisations determine FCI [54]. The reduction of scatter from R${}_{\varepsilon }=-1$ to R${}_{\varepsilon }=0$ at a constant total strain amplitude may be traced back to the intrinsically higher loading level of the microstructure at R${}_{\varepsilon }=0$. The resulting localization of plastic strains induced at R${}_{\varepsilon }=0$ is thereby less pronounced than at R${}_{\varepsilon }=-1$ and results in a decrease of FCI lifetime scatter.

## 5. Conclusions

In the present study, micromechanical fatigue crack initiation (FCI) predictions have been made for the martensitic high-strength steel SAE 4150 at R${}_{\varepsilon }=-1$ and R${}_{\varepsilon }=0$. Experimental investigations including strain-controlled fatigue crack initiation testing of the considered SAE 4150, EBSD analyses and light optical microscopy were used to characterize the fatigue crack initiation behaviour, to analyse the grain morphology and orientations as well as the underlying prior austenite grain size. Multiple martensitic microstructures incorporating the experimentally observed Nishiyama-Wassermann orientation relationship were generated for subsequent finite element modelling. In a previous study [31], an advanced phenomenological crystal plasticity model considering kinematic hardening by an Ohno Wang model was calibrated to the investigated material. In the present study, this model was used to investigate the fatigue crack initiation behaviour by means of three different non-local fatigue indicator parameters (FIP). A summary of the results is as follows:

- Martensitic microstructures exposed to fatigue loading exhibit strong local stress and strain heterogeneities due to inherent microstructural variations. These local inhomogeneities reveal the importance of micromechanical fatigue modelling techniques for fatigue crack initiation prediction in the low cycle fatigue regime as well as in the high cycle fatigue regime.
- A sphere homogenisation technique was introduced for the martensitic microstructure. Fatigue indicator parameters are averaged within a finite sphere in order to introduce an internal damage length scale. The averaged fatigue indicator parameters are termed as non-local fatigue indicator parameters.
- For fully reversed loadings R${}_{\varepsilon }=-1$, the three considered non-local FIPs (accumulated plastic slip, Fatemi-Socie and energy dissipation metric) show in general a good agreement to experimental fatigue crack initiation data. However, the three considered FIPs show increasing deviations with respect to fatigue crack initiation time for total strain amplitude less than $\varepsilon_{a,t}=0.40\%$. These deviations may be traced back to the increased level of plasticity of the constitutive model for small applied strains and the missing incorporation of microstructural short cracks. In future micromechanical simulations, the microstructural short crack growth has to be modelled in order to integrate further relevant mechanisms and stages of fatigue damage.
- Predictions of the fatigue crack initiation lifetime for R${}_{\varepsilon }=0$ indicate that the non-local accumulated plastic slip is not able to predict the influence of an increased strain ratio. The non-local Fatemi-Socie metric and the non-local energy dissipation criteria in principle enable the prediction of FCI lifetimes at increased strain ratios. Both criteria show a good qualitative agreement with experimental data for R${}_{\varepsilon }=0$, whereby the non-local Fatemi-Socie criteria is superior to the non-local energy dissipation metric. However, there are deviations for small applied strain amplitudes in the lifetime reduction factor characteristics. In analogy to the simulations at R${}_{\varepsilon }=-1$, the root cause for these deviations are in the overestimations of the plasticity level of the micromechanical model for small applied strains. The lifetime results of the non-local Fatemi-Socie and the non-local dissipated energy metric indicate a good transferability between different applied total strain ratios and enables thereby the prediction of fatigue crack initiation for arbitrary total strain ratios. In future, small strain amplitudes should be more focused during the constitutive model calibration process in order to avoid the overestimation of the level of plasticity.

## Figures and Tables

**Table 1:** Chemical composition of the SAE 4150 in wt.%.

Material	C	Si	Mn	P	S	Cr	Mo
SAE 4150	0.52	0.26	0.74	0.014	0.008	1.31	0.18

**Table 2:** Parameters of the Basquin-Manson-Coffin approach of Equation (1) for SAE 4150 at R${}_{\varepsilon }$ = −1 and ASTM A723 at R${}_{\varepsilon }$ = 0.

Material	$R_{\varepsilon }$	$\sigma_{f}^{{}^{\prime }}$	*E*	*b*	$\varepsilon_{f}^{{}^{\prime }}$	*c*	No. of Specimens
SAE 4150	−1	1862.0 MPa	208 GPa	−0.095	0.542	−0.708	18
ASTM A723	0	2650.0 MPa	200 GPa	−0.180	0.325	−0.620	6

**Table 3:** Morphological properties of the considered statistical volume elements.

Descriptor	SVE1	SVE2	SVE3	SVE4
Number of PAGs	7	6	6	8
Number of packets	5	6	5	6
Number of blocks	166	205	144	198

**Table 4:** Simulation environment and time consumption for one microstructural simulation.

Total number of elements:	125,000
Total number of nodes:	132,651
CPU:	Intel(R) Xeon(R) @ 2.60 GHz
Number of parallel CPUs:	24
Time consumption at R${}_{\varepsilon }$ = −1:	23 h
Time consumption at R${}_{\varepsilon }$ = 0:	86 h

**Table 5:** Set of crystal plasticity parameters for SAE 4150 (39 HRC) at room temperature [31].

Elastic Constants and Flow Parameters			Hardening Parameters		
**Parameter**	**Dimension**	**Value**	**Parameter**	**Dimension**	**Value**
$C_{11}$	GPa	253.1	A	GPa	65.506
$C_{22}$	GPa	132.4	B	-	499
$C_{44}$	GPa	75.8	M	-	8
${\dot{\gamma }}_{0}$	s${}^{-1}$	0.001			
$\tau_{c,0}^{\alpha }$	MPa	209			
*m*	-	100			

**Table 6:** Local and non-local critical fatigue indicator parameters for SAE 4150.

Spatial FIP Resolution	FIP${}_{crit,p}$	FIP${}_{crit,FS}$	FIP${}_{crit,W}$ $\left(\frac{\mathrm{MJ}}{m^{3}}\right)$
local (integration point values)	8.5 ×10	3.1 $\times \;10^{2}$	3.9 $\times \;10^{6}$
non-local (volume-averaged)	7.7 ×10	2.5 $\times \;10^{2}$	3.2 $\times \;10^{6}$

## Nomenclature

ASTM	American Society for Testing and Materials
BCC	Body Centred Cubic
CP	Crystal Plasticity
EBSD	Electron Backscatter Diffraction
FCI	Fatigue Crack Initiation
FIP	Fatigue Indicator Parameter
${\mathrm{FIP}}_{\mathrm{crit}}$	Critical Fatigue Indicator Parameter
${\mathrm{FIP}}_{\mathrm{cyc}}^{*}$	Non-Local Saturated Fatigue Indicator Parameter
FIP${}_{\mathrm{FS}}$	Fatemi-Socie Metric
FIP${}_{p}$	Accumulated Plastic Slip
FIP${}_{W}$	Energy Dissipation Criteria
HCF	High Cycle Fatigue
HRC	Rockwell Hardness
LCF	Low Cycle Fatigue
LOM	Light Optical Microscopy
MSC	Microstructural Short Crack
NW-OR	Nishiyama-Wassermann Orientation Relationship
PAG	Prior Austenite Grain
PSC	Physically Short Crack
RVE	Representative Volume Element
SEM	Scanning Electron Microscopy
SVE	Statistical Volume Element
UMAT	User Material Subroutine
*A*	Kinematic Hardening Parameter
*b*	Fatigue Strength exponent
*B*	Kinematic Hardening Parameter
*c*	Fatigue Ductility exponent
$C_{ij}$	Stiffness Tensor Components
dt	Time Increment
dV	Volume Increment
E	Young’s Modulus
*k*	Parameter of the Fatemi-Socie Metric
*m*	Power Law Exponent
*M*	Kinematic Hardening Exponent
$N_{crit}$	Cycles Required to Initiate a Microcrack
$N_{i}$	Fatigue Crack Initiation Cycles
$\dot{p}$	Plastic Slip Increment
R${}_{\varepsilon }$	Total Strain Ratio
$V_{pz}$	Process Zone Volume for Fatigue Crack Initiation
$A^{e}$	Elastic Part of Tensor A
$A^{p}$	Plastic Part of Tensor A
F	Deformation Gradient
L	Velocity Gradient
$m^{\alpha }$	Unit Vector in Slip Direction
$n^{\alpha }$	Unit Vector normal to Slip Plane
α	Slip System
$\alpha^{\prime }$	Martensite Phase
$\gamma^{\prime }$	Austenite Phase
${\dot{\gamma }}^{\alpha }$	Shear Rate of Slip System α
${\dot{\gamma }}_{0}$	Reference Shear Rate
$\gamma_{p}$	Local Plastic Shear
Γ	Lifetime Reduction Factor
Δa	Delta Of Quantity *a*
$\varepsilon_{a,el}$	Elastic Strain Amplitude
$\varepsilon_{a,pl}$	Platic Strain Amplitude
$\varepsilon_{a,t}$	Total Strain Amplitude
$\varepsilon_{t,min}$	Minimum Total Strain
$\varepsilon_{t,max}$	Maximum Total Strain
$\varepsilon_{f}^{{}^{\prime }}$	Fatigue Ductility Coefficient
$\sigma_{f}$	Yield Strength
$\sigma_{u}$	Ultimate Tensile Strength
$\sigma_{f}^{{}^{\prime }}$	Fatigue Strength Coefficient
$\sigma_{ij}$	Stress Tensor Components
$\sigma_{n}^{\alpha }$	Normal Stress of Slip System α
$\tau^{\alpha }$	Resolved Shear Stress of Slip System α
$\tau_{c,0}$	Critical Resolved Shear Stress
$\chi_{b}^{\alpha }$	Resolved Back Stress of Slip System α
${\dot{\chi }}_{b}^{\alpha }$	Resolved Back Stress Increment of Slip System α
